# Ketamine and metabolites in snake venom: effects of venom extraction and potential impact on animal models

**DOI:** 10.1038/s41598-025-32525-6

**Published:** 2025-12-16

**Authors:** Kerstin Damm, Christoph Hartwig, Andreas Vilcinskas, Stephen P. Mackessy, Tim Lüddecke, Maik Damm

**Affiliations:** 1 Justus-Liebig-University Giessen, Giessen, Germany; 2 Branch for Bioresources, Fraunhofer Institute for Molecular Biology and Applied Ecology (IME), Ohlebergsweg 12, 35392 Giessen, Germany; 3 Institute of Insect Biotechnology, Justus-Liebig-University Giessen, Heinrich-Buff-Ring 26–32, 35392 Giessen, Germany; 4 LOEWE Centre for Translational Biodiversity Genomics (LOEWE-TBG), Senckenberganlage 25, 60325 Frankfurt am Main, Germany; 5 Department of Biological Sciences, University of Northern Colorado, Greeley, CO 80639 USA; 6 Animal Venomics Lab, Fraunhofer Institute for Molecular Biology and Applied Ecology IME, Ohlebergsweg 12, 35392 Giessen, Germany

**Keywords:** Metabolism, Drugs, Quantification, Mass spectrometry, Biochemistry, Biological techniques, Chemical biology, Drug discovery, Zoology

## Abstract

In zootoxinology, drugs such as anesthetics and secretion enhancers are used to increase venom yields, but it is unclear whether this affects the venom composition. After injection of ketamine and pilocarpine into two non-front-fanged snakes, mass spectrometry confirmed the presence of both drugs and their metabolites in the venoms. The quantified high concentrations raise concerns about potential interference in bioassays and pharmacological studies, highlighting the need to consider extraction additives in venom research to ensure unbiased results.

**Supplementary Information:**

The online version contains supplementary material available at 10.1038/s41598-025-32525-6.

## Introduction

 The study of animal toxins (zootoxinology) lies at the interface of important aspects of zoology, biochemistry, and biomedicine^1^. Venoms (transmitted by active injection) and poisons (by topical contact) are complex mixtures containing up to thousands of different toxins. Venoms are excellent models to answer fundamental biological questions in evolution and structure/function studies, and to generate valuable bioresources^2^. Activities are classically tested by in vivo animal models, e.g. lethal dose (LD_50_) determination in mice. Novel strategies, such as bioactivity screening or pharmacological studies, are based on various in vitro analytical methods, termed Modern Venomics, and a key element is the extraction of venoms^3^.

Various non-lethal approaches have been developed to extract venom from animals. Manual extraction is easily feasible for larger animals, such as snakes, while smaller organisms, e.g. most spiders and scorpions, may require electrostimulation. However, many venom systems cannot be easily accessed and chemical extraction additives are needed to stimulate the secretion of venoms and aid their collection^3^. Non-front-fanged snakes are one such example. This group of snakes lacks the characteristic enlarged fangs at the anterior maxilla. Instead, are either located on the posterior upper jaw or are not present at all. Further, in contrast to other venomous snakes, they contain Duvernoy’s venom glands that often produce low venom amounts compared to venom glands of front-fanged snakes. Hence, their venoms and venom delivery systems are highly understudied^4^. For decades, sedation with ketamine, followed by stimulation of venom release by pilocarpine, a muscarinic receptor agonist, has been a common practice^5,6^. This approach increases the venom amount without altering its composition^7^.

## Results

**Fig. 1:**
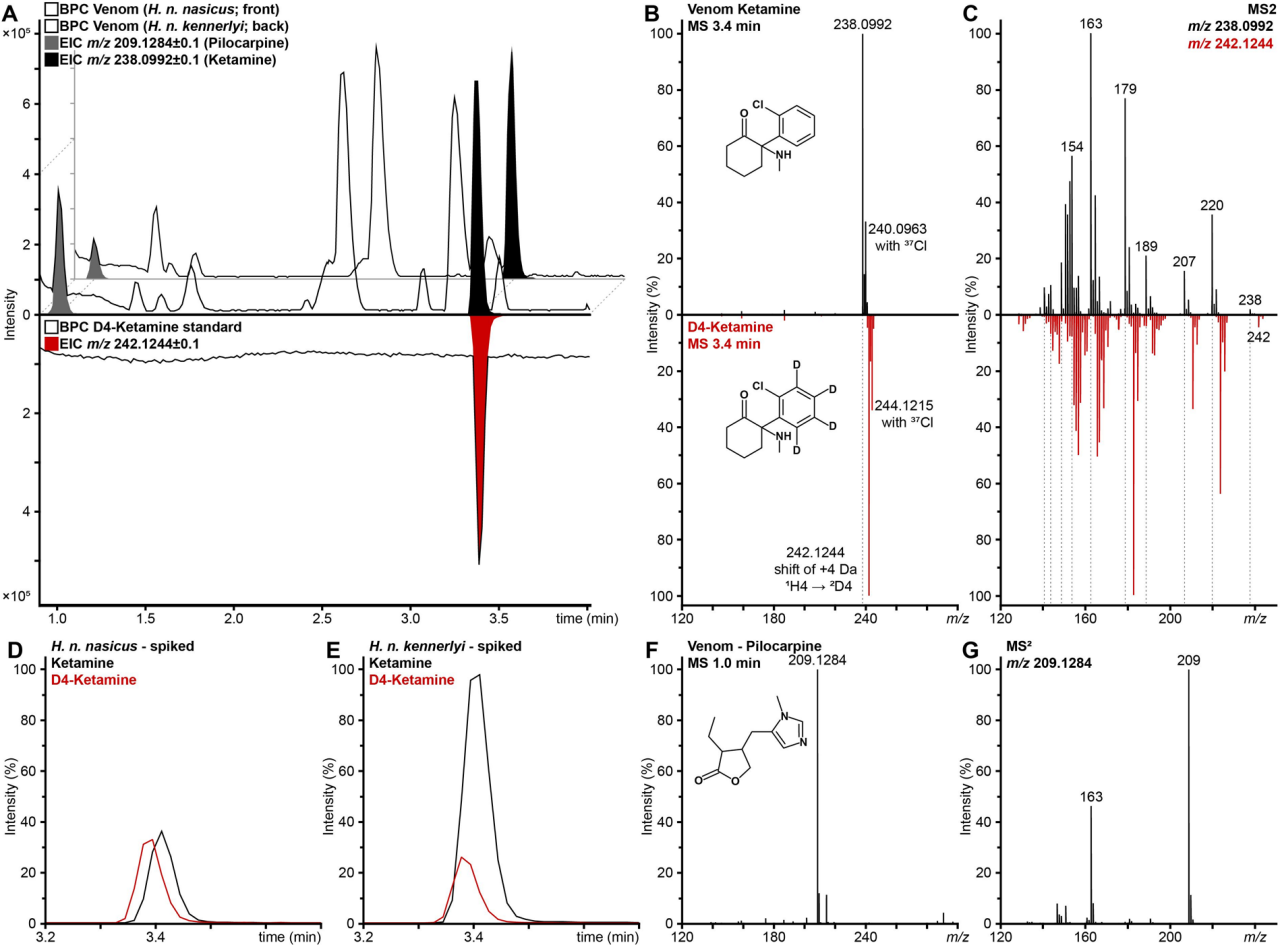
Identification and quantification of small molecules in snake venoms. (**A**) Base peak chromatograms (BPC) of two snake venoms and D_4_-ketamine (certified reference standard) separated by C18-RP HPLC at early retention times, with the extracted ion chromatograms (EIC) of protonated pilocarpine (grey), ketamine (black) and isotopically-labeled D_4_-ketamine (red). (**B**) Structure, MS and (**C**) MS^2^ comparison of in-venom and standard D_4_-ketamine, with isotope mass shift (dotted line). Internal EIC quantification of ketamine (black) versus spiked reference standard D_4_-ketamine (0.5 ng/µL) in (**D**) *H. n. nasicus* and (**E**) *H. n. kennerlyi* venom. (**F**) Structure, MS and (**G**) MS^2^ of pilocarpine in venom; data shown for *H. n. nasicus*.

During our studies of snake venom compositions, we detected an abundant signal of *m/z* 238 with peptide-atypical isotope pattern and tandem MS (MS^2^) fragmentation in two *Heterodon nasicus ssp.* venoms (Fig. 1A-C; top). Manual *de novo* annotation and comparison to spectra from the literature confirmed the ion mass as ketamine, with its chlorine (^35^Cl and ^37^Cl, ratio 76:24) responsible for the peptide-atypical signal^8^. Therefore, isotopically-labeled D_4_-ketamine hydrochloride was subsequently measured for identification and quantification. The certified reference showed comparable retention times, isotope distribution and fragmentation retrospective to the expected +4 Da shift stemming from the four deuterium atoms (Fig. 1A-C; red). Therefore, we confirmed that the injected ketamine was distributed into the snake venom, as this synthetic drug cannot originate from the animal itself. For quantification, D_4_-ketamine was added as an internal standard and its area under the curve compared to the venom-derived ketamine (Fig. 1D, E). They contained 30 µM and 121 µM ketamine with injection-to-venom recovery rates of 0.02% (m/m) and 0.09% (m/m), respectively for *H. n. nasicus* and *H. n. kennerlyi*.

Next, the MS data were screened for the also injected pilocarpine. Its mass (*m/z* 209) was found and its identity verified on MS and MS^2^ levels (Fig. 1A, F, G). Since both substances have known metabolic pathways, we hypothesized that some could be found in the venom. In total, eight metabolites were detectable with abundances lower than the non-metabolized precursors (Table 1, S1, Figure S1). All substances were identified in both venoms by their exact mass and isotope pattern via high-resolution MS and, except phenol-hydroxy-/dihydroxy-norketamine, by MS^2^ fragmentation (Fig. 1C, G, S2-S11).

**Table 1:** Identified substances and metabolites in snake venom after subcutaneous injection of ketamine and pilocarpine to *Heterodon nasicus nasicus* for venom milking.

Substance	Ion M + H^+^ sum formula	*m/z* M + H^+^ theo. mass	*m/z* M + H^+^ obs. mass	Mass error in ppm	mSigma	Tandem-MS confirmation	Isotope ratio ^37^Cl:^35^Cl
Ketamine	C_13_H_17_ClNO	238.0993	238.0994	-0.3	7.5	✓	32%
Phenol-ketamine	C_13_H_17_ClNO_2_	254.0942	254.0945	-1.0	77.6	✓	32%
Hydroxy-ketamine	C_13_H_17_ClNO_2_	254.0942	254.0942	0.3	40.6	✓	26%
Norketamine	C_12_H_15_ClNO	224.0837	224.0838	-0.6	42.1	✓	26%
Phenol-norketamine	C_12_H_15_ClNO_2_	240.0786	240.0789	-1.2	30.7	✓	29%
Hydroxynorketamine	C_12_H_15_ClNO_2_	240.0786	240.0789	-1.3	20.2	✓	30%
Dehydronorketamine	C_12_H_13_ClNO	222.0680	222.0680	0.2	51.6	✓	26%
Phenol-hydroxy-/ dihydroxy-norketamine	C_12_H_15_ClNO_3_	256.0741	256.0734	0.6	n.a.	-	37%
Pilocarpine	C_11_H_17_N_2_O_2_	209.1285	209.1286	-0.6	3.9	✓	n.a.
Pilocarpic acid	C_11_H_19_N_2_O_3_	227.1390	227.1393	-1.1	2.4	✓	n.a.

## Discussion

These findings should raise awareness that extraction additives and their metabolites can be sequestered into the venom following their injection. This may have functional repercussion, since ketamine, an NMDAR antagonist, and its metabolites, such as norketamine and hydroxy(nor)ketamine, interact with a variety of receptors, ion channels and pathways^9^. The second substance, pilocarpine, is a muscarinic, cholinergic agonist affecting smooth muscles, exocrine tissue, and Ca^2+^ response^10^. Ca^2+^ and related channels are important cellular regulators and prominent targets of animal toxins^11,12^. Therefore, the presence of these additives in the analyzed venom has uncertain potential to interfere with downstream experiments, particularly pharmacokinetics, bioactivities or even toxicity assays. In addition, adrenaline, serotonin and dopamine have been used in zootoxinology to increase venom yields^13^, but only one study has investigated this transition^14^. They inferred a 0.8–23% (5–53 mM) pilocarpine recovery from tick venom following injection.

Regarding the pharmacokinetics, ketamine is a highly potent substance, affecting e.g. N-methyl-d-aspartate receptors (NMDAR; IC_50_ 0.35-10 µM), intracellular d-serine increase (IC_50_ 0.70 µM, EC_50_ 0.76 µM), GABA uptake (IC_50_ 50 µM) and various cholinergic receptors at micromolar concentrations^9^. Our determined 30–121 µM ketamine concentrations are in a range high enough to potentially effect receptors/targets in rat and mouse models^9^. Although rodents have historically been favored, other animals such as zebrafish (*Danio rerio*), fruit fly (*Drosophila melanogaster*), and shrimp (*Artemia salina*) have been used as test systems in zootoxinology^3,15^. However, it is unknown how most animal models and novel alternative replacements react to the extraction additives and their metabolites in combination with venom. For instance, lowest concentrations of ketamine and other drugs in forensic entomotoxicology affected the development of blowflies (*Lucilia*, *Chrysomya*)^16,17^. In addition to in vivo effects, in vitro experiments may also be affected, as organ-on-a-chip models, cell cultures, *Xenopus* oocytes and electrophysiological studies are highly sensitive. Adding parasympathomimetics (pilocarpine), receptor antagonists (ketamine) or hormones (adrenaline, dopamine, serotonin) should be considered as potential trigger for unknown positive or negative feedback.

These considerations are theoretical and require further investigation. Our results represent a preliminary step into this novel topic, but are limited to just two male specimens of a single species of non-front-fanged snake with a large Duvernoy’s gland. If the observation are intraspecific or individual variations are unknown at this point and should also be considered in future studies, including larger sample sizes, both sexes and the venom of untreated snakes as negative control. To our knowledge, this is the first study of this phenomenon in snakes and the second in venomous animals (ticks, Ixodidae) ever reported^14^. It raises the question of whether this effect can be generalized, as observed in two distinct classes of Reptilia and Arachnida in different phyla, and should be investigated in future studies also for other taxa.

Our findings show the field of zootoxinology should increase their awareness about the possibility that venom extraction additives can contaminate venom samples at pharmacologically relevant concentrations and hence may cause artefacts in bioactivity profiling experiments. Future studies that include this practice should rule out the potential for cross-reaction between the additives and their metabolites in the venom and the system being investigated. The impact on current and future animal models remains uncertain. Although the substances involved are highly bioactive at millimolar concentrations, further analyses are required to confirm or rule out whether the described process introduces bias in the resulting data. Additionally, it would be interesting to test (historical) collections for their appearance, as most of them remain stable during long-term storage.

## Methods

### Animals

Two male snakes (*Heterodon nasicus ssp.)* were collected in the wild; both were mature adults (approx. 8–9 years old). The Western Hognose Snake (*H. n. nasicus*) was collected in Weld Co., Colorado (permit 18HP0974, Colorado Parks and Wildlife). The Mexican Hognose Snake (*H. n. kennerlyi*) was collected in Hidalgo Co., New Mexico (permit 3418, New Mexico Department of Game and Fish). Both scientific collecting permits were issued to SPM. Snakes were housed individually and maintained at the UNC Animal Resource Facility until sacrifice; all procedures were previously reviewed and approved by the UNC Institutional Animal Care and Use Committee (protocol #2303D-SM-S-26). Additionally, animal care and use followed guidelines published by the American Society of Ichthyologists and Herpetologists (2004)^18^, and methods used are in accordance with ARRIVE guidelines.

Snakes (*H. n. nasicus*, 186.9 g; *H. n. kennerlyi*, 129.0 g) were immobilized using subcutaneously injected ketamine HCl (45 µg/g) and kept in a warm, dark room. Approx. 15 min later, subcutaneously administered pilocarpine HCl (6 µg/g) was used to stimulate venom secretion^6^. Approx. 5–10 min later, venom flow began and was collected in 100 µL capillary tubes placed over the enlarged rear maxillary fangs. Venom was transferred to Eppendorf tubes on ice, centrifuged at 9,000 × g for 5 min to pellet solids, frozen at -80 °C and then lyophilized. Lyophilized venom was stored at -20 °C. In total, the snakes gave 160 µL (*H. n. nasicus*) and 150 µL (*H. n. kennerlyi*) crude venom, resulting in 5.0 mg lyophilized venom, each, representing an expected quantity for these subspecies (SPM, personal experience).

### Mass spectrometry

Lyophilized venom (100 µg) was resolved in 50 µL aqueous buffer (30 mM citrate buffer (pH 3), 0.2% (v/v) formic acid) and centrifuged at 12,000 × g for 10 min to pellet solids. Identically, a blank (30 mM citrate buffer (pH 3), 0.2% (v/v) formic acid) without venom was prepared. Samples (5 µL) and blank (5 µL) were loaded to a reversed-phase ACQUITY UPLC Peptide BEH C18 (300 Å, 1.7 μm, 2.1 mm × 100 mm) column with the appropriate pre-column (ACQUITY UPLC Peptide BEH C18 VanGuard Pre-column, 300Å, 1.7 μm, 2.1 mm × 5 mm) and chromatographically separated with an Agilent 1290 Infinity LC system (Agilent Technologies). The following gradient with ultrapure water with 0.1% (v/v) formic acid (solvent A) and acetonitrile with 0.1% (v/v) formic acid (solvent B) was used at 0.6 mL/min, with a linear gradient between the time points, given at min (B%): 0–0.3 (5% const.), 0.3–18 (5 to 95%), 18–18.1 (95 to 100%), 18.1– 22.5 (100% const.), and 2.5 min re-equilibration at 5%. MS analyses were carried out on an ESI QTOF maXis II mass spectrometer (Bruker Daltonics) in positive mode. Data were acquired in the scan range *m/z* 50–2000. MS/MS analysis was performed for the top five most intense ions (30 s exclusion list) selected for collisional induced dissociation (CID) using N_2_.

### Ketamine quantification

Isotopically labelled and certified reference D_4_-ketamine HCl (100 ng/µL, Cerilliant^®^; Supelco) were diluted (1:20) to 5 ng/µL in ultra-pure water. For spiking, 100 µg venom were processed as in Methods – Mass spectrometry, with the change of a volume of 45 µL venom solution to add 5 µL (25 ng) diluted D_4_-ketamine HCl, reaching 50 µL final volume (60 mM citrate buffer (pH 3), 0.2% (v/v) formic acid). Spiked venoms were measured as descripted in Methods – Mass spectrometry. Area under the curve of the extracted ion chromatograms for ketamine (*m/z* 238.0993 ± 0.01) and D_4_-ketamine (*m/z* 242.1244 ± 0.01) were determined with DataAnalysis (Compass DataAnalysis; Bruker Daltonics, Version 5.3).

## Supplementary Information

Below is the link to the electronic supplementary material.

Supplementary Material 1

## Data Availability

Data supporting the findings of this study are available within the paper and its Supplementary Information. Raw data that support the findings of this study are available upon request from the corresponding authors.
