# Supplementary material for: Ketamine and metabolites in snake venom: effects of venom extraction and potential impact on animal models
Source: Sci Rep. 2025 Dec 16;15:43950. doi: 10.1038/s41598-025-32525-6 (PMC12712038; doi:10.1038/s41598-025-32525-6)
Supplement: Supplementary file 1 — Supplementary Material 1 [file 41598_2025_32525_MOESM1_ESM.pdf]

# – Supplementary Information –

Ketamine and Metabolites in Snake Venom: Effects of Venom Extraction and Potential Impact on Animal Models

Kerstin Damm [1,#], Christoph Hartwig [2], Andreas Vilcinskas [2,3,4], Stephen P. Mackessy [5], Tim Lüddecke [4,6], Maik Damm [3,4,6,#,*]

1 Justus-Liebig-University Giessen, Germany

2 Branch for Bioresources, Fraunhofer Institute for Molecular Biology and Applied Ecology (IME), Ohlebergsweg 12, 35392 Giessen, Germany

3 Institute of Insect Biotechnology, Justus-Liebig-University Giessen, Heinrich-Buff-Ring 26–32, 35392 Giessen, Germany

4 LOEWE Centre for Translational Biodiversity Genomics (LOEWE-TBG), Senckenberganlage 25, 60325 Frankfurt am Main, Germany

5 Department of Biological Sciences, University of Northern Colorado, Greeley, CO USA 80639

6 Animal Venomics Lab, Fraunhofer Institute for Molecular Biology and Applied Ecology IME, Ohlebergsweg 12, 35392 Giessen, Germany

*shared first author; *Corresponding author: maik.damm@agrar.uni-giessen.de*

Table S1: Identified substances and metabolites in snake venom after subcutaneous injection of ketamine and pilocarpine to *Heterodon nasicus kennerlyi.* for venom milking.

Substance	Ion Sum formula	*m/z* M+H⁺ theo. mass	*m/z* M+H⁺ obs. mass	Mass error in ppm	mSigma	Tandem-MS confirmation	Isotope ratio ^{37}Cl:^{35}Cl
ketamine	$C_{13}H_{17}ClNO$	238.0993	238.0994	-0.3	13.2	✓	32%
phenol-ketamine	$C_{13}H_{17}ClNO_2$	254.0942	254.0943	-0.2	77.8	✓	35%
hydroxy-ketamine	$C_{13}H_{17}ClNO_2$	254.0942	254.0945	-1.1	25.3	✓	35%
norketamine	$C_{12}H_{15}ClNO$	224.0837	224.0838	-0.8	22.9	✓	34%
phenol-norketamine	$C_{12}H_{15}ClNO_2$	240.0786	240.0792	-2.5	44.0	✓	26%
hydroxynorketamine	$C_{12}H_{15}ClNO_2$	240.0786	240.0789	-1.1	25.6	✓	29%
dehydronorketamine	$C_{12}H_{13}ClNO$	222.0680	222.0679	-1.9	37.0	✓	35%
phenol-hydroxy-/ dihydroxy-norketamine	$C_{12}H_{15}ClNO_3$	256.0741	256.0735	-2.3	n.a.	-	42%
pilocarpine	$C_{11}H_{17}N_2O_2$	209.1285	209.1287	-1.4	8.1	✓	n.a.
pilocarpic acid	$C_{11}H_{19}N_2O_3$	227.1390	227.1394	-1.5	17.9	✓	n.a.

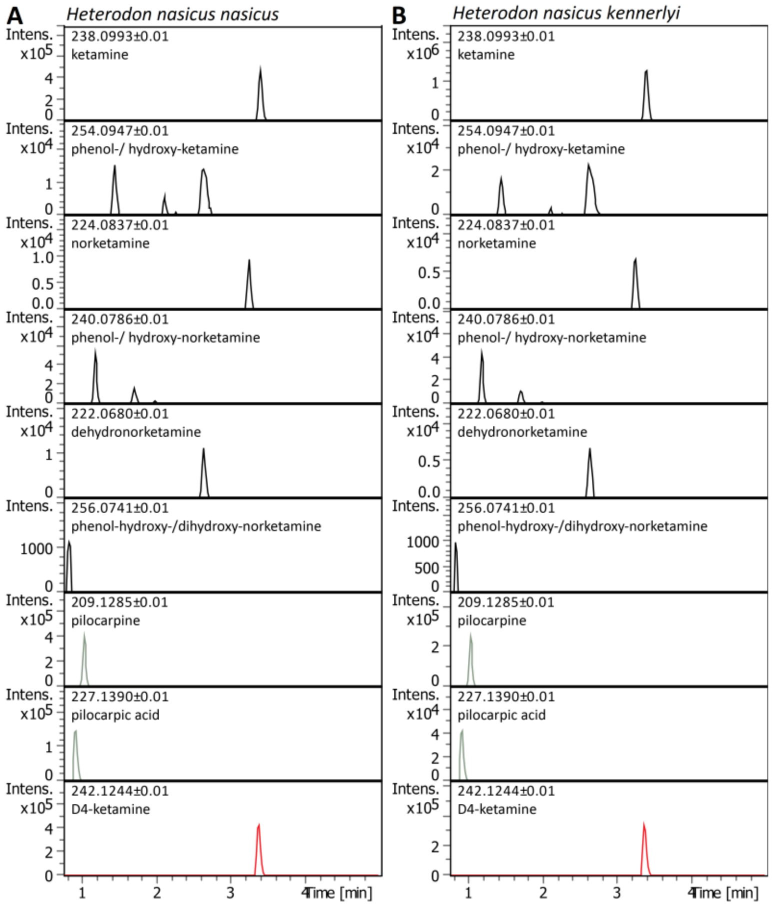

Figure S1: BPC and EIC of extraction additive contaminated snake venom. Base signal chromatograms (BPC) of **(A)** *Heterodon nasicus nasicus* and **(B)** *Heterodon nasicus kennerlyi* with spiked D_4-ketamine certified reference standard separated by C18-RP HPLC at early retention times, with the extracted ion chromatograms (EIC) of ketamine (black), pilocarpine (grey), their metabolites and isotopically-labeled D_4-ketamine (red).

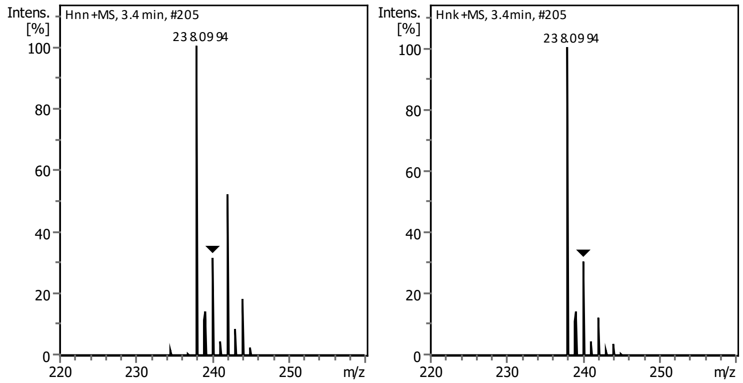

Figure S2: MS of ketamine in spiked snake venom. Intact mass of ketamine (theo. mass *m/z* 238.0993) in spiked *Heterodon nasicus nasicus* (left) and *Heterodon nasicus kennerlyi* (right) venom. Related ^{37}Cl signal marked by triangle.

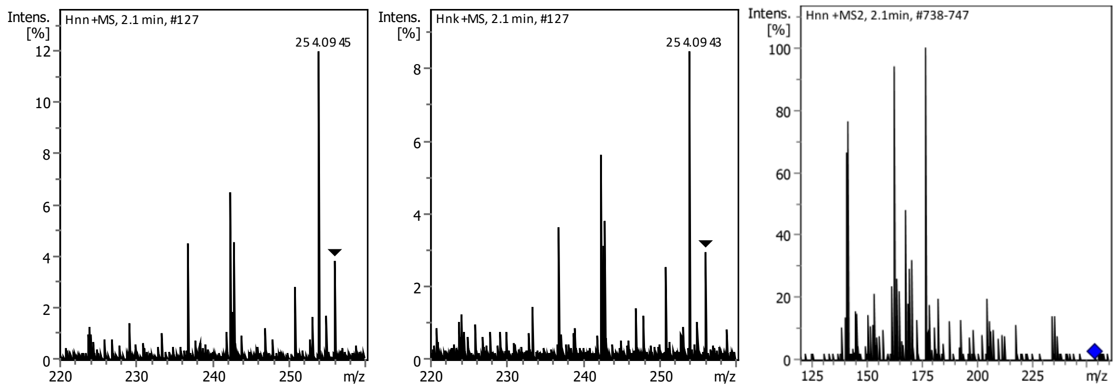

Figure S3: MS and MS² of phenol-ketamine in spiked snake venom. Intact mass of phenol-ketamine (theo. mass *m/z* 254.0947) in spiked *Heterodon nasicus nasicus* (left) and *Heterodon nasicus kennerlyi* (middle) venom. Related ^{37}Cl signal marked by triangle. Exemplary MS² in *H. n. nasicus* venom (right), with precursor ion marked by blue diamond.

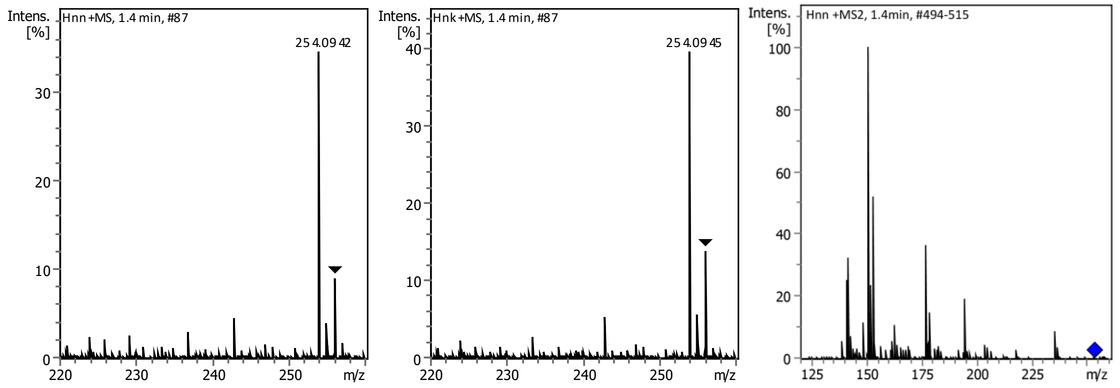

Figure S4: MS and MS² of hydroxy-ketamine in spiked snake venom. Intact mass of hydroxy-ketamine (theo. mass *m/z* 254.0947) in spiked *Heterodon nasicus nasicus* (left) and *Heterodon nasicus kennerlyi* (middle) venom. Related ^{37}Cl signal marked by triangle. Exemplary MS² in *H. n. nasicus* venom (right), with precursor ion marked by blue diamond.

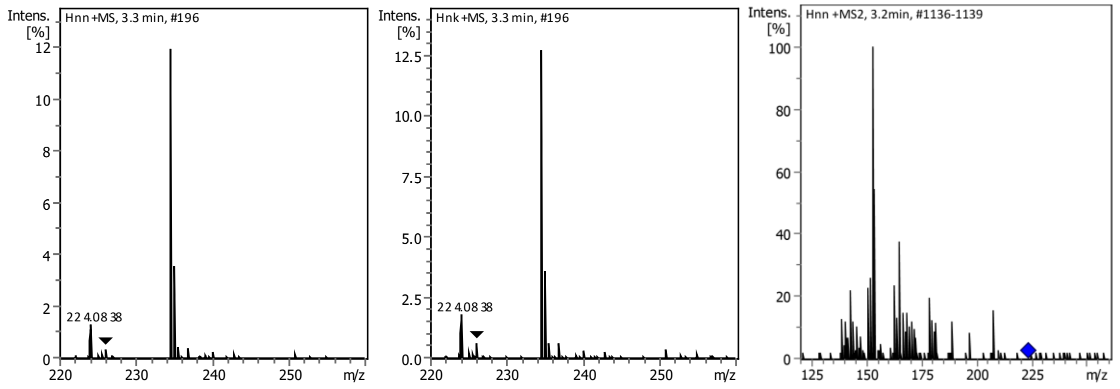

Figure S5: MS and MS² of norketamine in spiked snake venom. Intact mass of norketamine (theo. mass *m/z* 224.0837) in spiked *Heterodon nasicus nasicus* (left) and *Heterodon nasicus kennerlyi* (middle) venom. Related ^{37}Cl signal marked by triangle. Exemplary MS² in *H. n. nasicus* venom (right), with precursor ion marked by blue diamond.

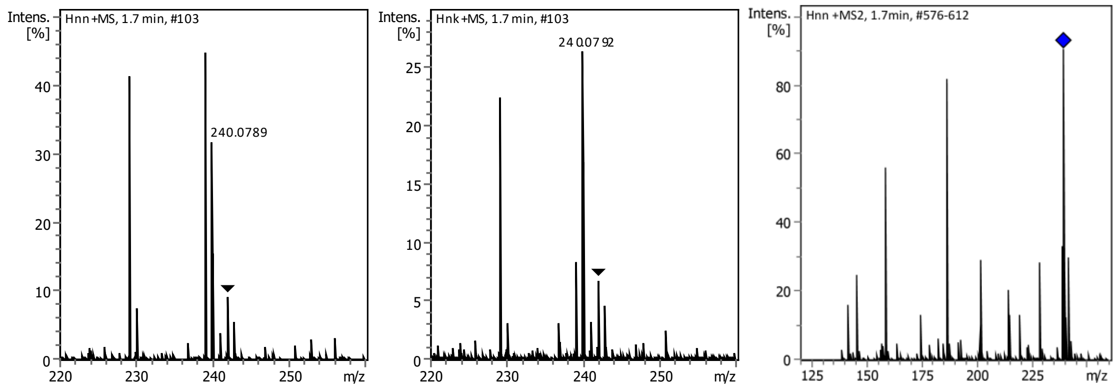

Figure S6: MS and MS² of phenol-norketamine in spiked snake venom. Intact mass of phenol-norketamine (theo. mass *m/z* 240.0786) in spiked *Heterodon nasicus nasicus* (left) and *Heterodon nasicus kennerlyi* (middle) venom. Related ^{37}Cl signal marked by triangle. Exemplary MS² in *H. n. nasicus* venom (right), with precursor ion marked by blue diamond.

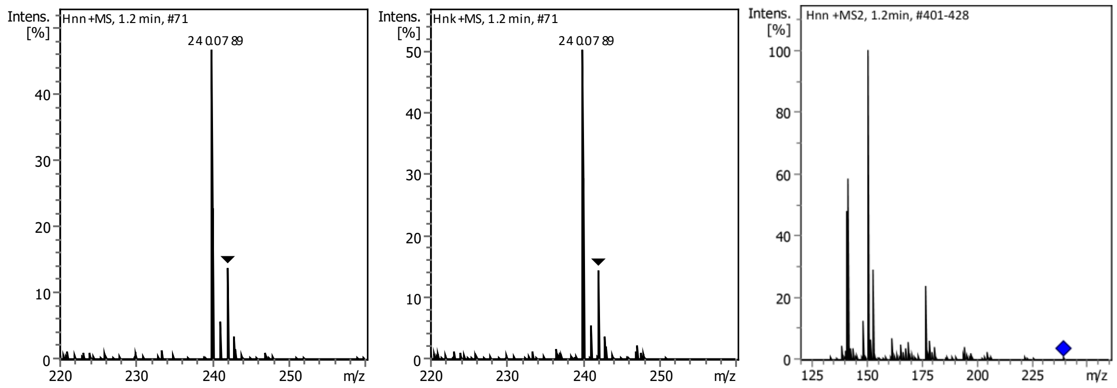

Figure S7: MS and MS² of hydroxynorketamine in spiked snake venom. Intact mass of hydroxynorketamine (theo. mass *m/z* 240.0786) in spiked *Heterodon nasicus nasicus* (left) and *Heterodon nasicus kennerlyi* (middle) venom. Related ^{37}Cl signal marked by triangle. Exemplary MS² in *H. n. nasicus* venom (right), with precursor ion marked by blue diamond.

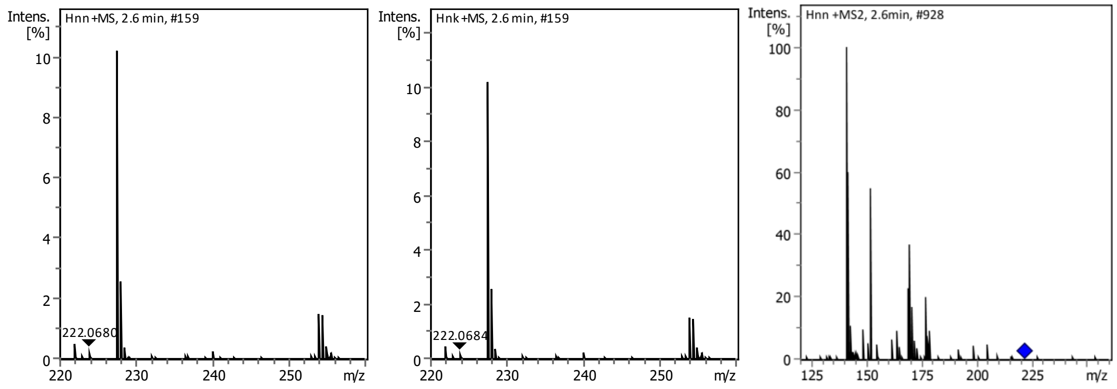

Figure S8: MS and MS² of dehydronorketamine in spiked snake venom. Intact mass of dehydronorketamine (theo. mass *m/z* 222.0680) in spiked *Heterodon nasicus nasicus* (left) and *Heterodon nasicus kennerlyi* (middle) venom. Related ^{37}Cl signal marked by triangle. Exemplary MS² in *H. n. nasicus* venom (right), with precursor ion marked by blue diamond.

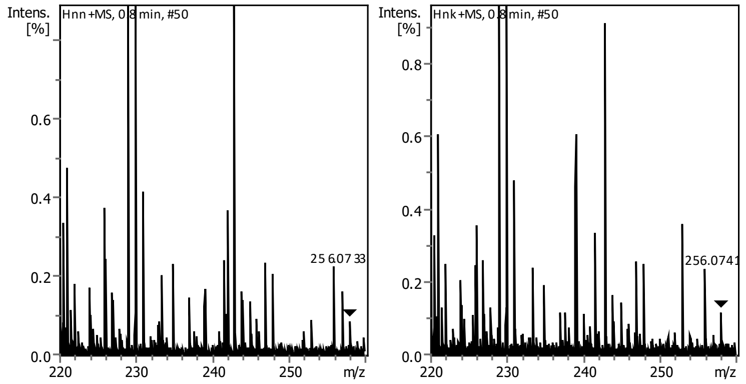

Figure S9: MS of phenol-hydroxy-/dihydroxy-norketamine in spiked snake venom. Intact mass of phenol-hydroxy-/dihydroxy-norketamine (theo. mass *m/z* 256.0741) in spiked *Heterodon nasicus nasicus* (left) and *Heterodon nasicus kennerlyi* (right) venom. Related ^{37}Cl signal marked by triangle.

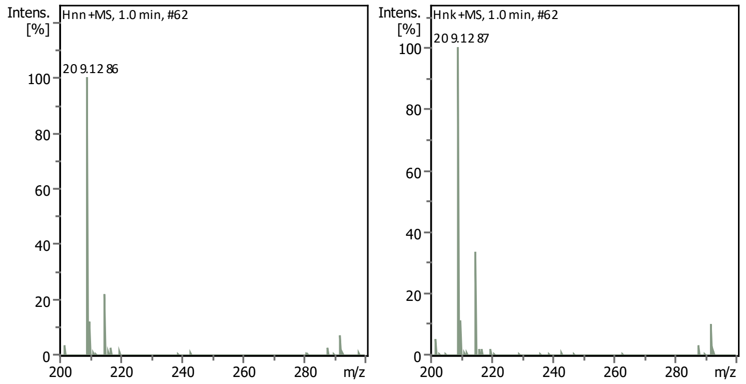

Figure S10: MS of pilocarpine in spiked snake venom. Intact mass of pilocarpine (theo. mass *m/z* 209.1285) in spiked *Heterodon nasicus nasicus* (left) and *Heterodon nasicus kennerlyi* (right) venom.

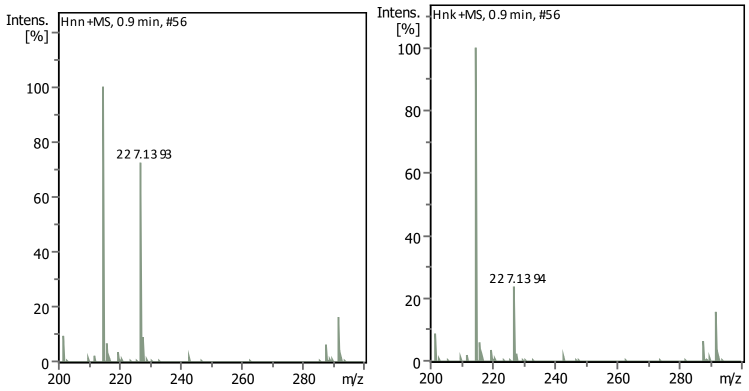

Figure S10: MS of pilocarpic acid in spiked snake venom. Intact mass of pilocarpic acid (theo. mass *m/z* 227.1390) in spiked *Heterodon nasicus nasicus* (left) and *Heterodon nasicus kennerlyi* (right) venom.

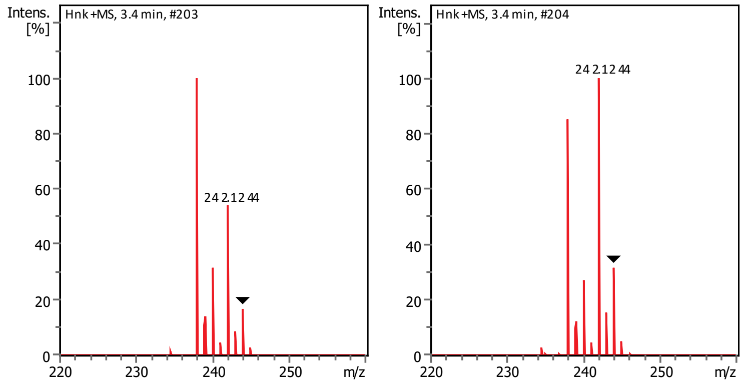

Figure S10: MS of spiking D^4-ketamine in the spiked snake venom. Intact mass of the D^4-ketamine (theo. mass *m/z* 242.1244) in spiked *Heterodon nasicus nasicus* (left) and *Heterodon nasicus kennerlyi* (right) venom. Related ^{37}Cl signal marked by triangle.
